# An innovative safe anesthesia and analgesia package for emergency pediatric procedures and surgeries when no anesthetist is available

**DOI:** 10.1186/s12245-016-0113-8

**Published:** 2016-06-10

**Authors:** Kevin R. Schwartz, Karla Fredricks, Zaid Al Tawil, Taylor Kandler, Stella A. Odenyo, Javan Imbamba, Brett D. Nelson, Thomas F. Burke

**Affiliations:** Division of Global Health and Human Rights, Department of Emergency Medicine, Massachusetts General Hospital, 125 Nashua Street, Suite 910, Boston, MA 02114 USA; Harvard Medical School, Boston, MA 02115 USA; African Institute for Health Transformation, Sagam Community Hospital, Luanda, Kenya

**Keywords:** Ketamine, Pediatric analgesia, Anesthesia, Low-income, Resource-limited, Sub-Saharan Africa, Non-anesthetist

## Abstract

**Background:**

Adequate pain control through sedation and anesthesia for emergency procedures is a crucial aspect of pediatric emergency care. Resources for administering such anesthesia are extremely limited in many low-income settings.

**Methods:**

Non-anesthetist providers in Western Kenya were trained in the use of a ketamine-based sedation and anesthesia package for non-anesthetists, *Every Second Matters for Mothers and Babies-Ketamine™* (ESM-Ketamine). Data on use and safety of this package for emergent and urgent pediatric procedures was collected. Providers were surveyed as to what they would have done for similar procedures if the ESM-Ketamine package were unavailable.

**Results:**

Ninety procedures were completed for 77 pediatric patients utilizing the ESM-Ketamine package. Of these, 29 (32.2 %) cases were orthopedic reductions, 19 (21.1 %) were incision and drainage, and 19 (21.1 %) were debridement and irrigation of burns. Remaining cases included cesarean section, repair of perineal tear, foreign body removal, arthrocentesis, laceration repair, exploratory laparotomy, excision of mass, paracentesis, and circumcision. There were no serious adverse events in any of the cases, 17 % experienced minor adverse events including hypersalivation, hallucinations, or brief, self-resolving, oxygen desaturations. Providers were surveyed for 80 of the 90 cases as to what they would have done in the absence of the ESM-Ketamine package: in 26 cases (32.5 %), they reported they would proceed with the procedure without any anesthesia or analgesia; in 15 (18.75 %), they reported they would significantly delay the procedure while waiting for an anesthetist; in 13 (16.25 %), they reported they would attempt referral to another facility; and in 26 (32.5 %), they reported they would try using an alternate form of analgesia, primarily acetaminophen, ibuprofen, diclofenac, and/or diazepam. All surveyed providers reported they would use the ESM-Ketamine package again in similar cases.

**Conclusions:**

The ESM-Ketamine package, through the use of a simplified protocol and checklist, allows for safe analgesia and anesthesia in children by non-anesthetists in a resource-limited setting for selected emergent and urgent procedures. This package addresses a significant gap in the availability of anesthesia services in low-income settings that would otherwise result in significant delays to procedures or proceeding with painful procedures with inadequate analgesia.

## Background

Adequate pain control through analgesia, procedural sedation, and general anesthesia is a critical component of care for patients undergoing surgery and painful procedures [1]. This is particularly the case in children undergoing emergency procedures, where inadequate pain control may result in the inability to complete a procedure, untoward complications of the procedure, and significant distress to both child and parents [2].

Despite the widely recognized need for analgesia and anesthesia during surgery and painful procedures for adults and children, worldwide resources for administering such services are unevenly distributed and, in many regions, such as sub-Saharan Africa, are extraordinarily scarce [3]. The recent *Lancet* Commission on Global Surgery estimates that five of the 7.2 billion people on earth do not have access to emergency surgery and that lack of access to anesthesia is one of the primary barriers contributing to this situation [4]. This gap in the availability of safe anesthesia and analgesia in low- versus high-income countries has been recognized across a variety of geographic settings [5–7]. Some of the poorest countries in the world have the lowest density of anesthetists and anesthesiologists [5]. This anesthesia gap may be even more significant for children than for adult patients, as provider training and comfort with pediatric anesthesia and analgesia is especially uncommon in many low-income settings where anesthesia-related morbidity and mortality rates can be high [8–11].

Contributors to these negative outcomes are many, including unreliable electricity sources and oxygen delivery, inconsistent medication availability, inadequate facilities, and, in particular, a lack of personnel trained in the safe administration of sedation, anesthesia, and analgesia in children [3, 11]. This may result in painful urgent and emergent procedures in children being performed with inadequate pain control or being delayed for unacceptable amounts of time [12].

To address this anesthesia and analgesia gap for emergency surgery and procedures, we developed a pilot program, *Every Second Matters for Mothers and Babies-Ketamine™* (ESM-Ketamine), to train non-anesthetist providers in the administration and monitoring of ketamine anesthesia and sedation. Ketamine has an excellent demonstrated safety profile in pediatric patients, even in settings with limited resources [12–14]. Ketamine is inexpensive, widely available for distribution in sub-Saharan Africa, and a particularly well-suited choice to this setting as it provides both analgesic and dissociative effect [12]. In addition, significant respiratory depression and the need for ventilatory support, which can be challenging in settings where advanced airway experience and supplies are often scarce, occur extremely rarely with ketamine [15, 16].

Safety and effectiveness of the ESM-Ketamine package, designed for cesarean section and other emergency and painful life-improving procedures, has previously been reported in a series of 193 patients, 58 of whom were in the pediatric age group [17]. Here, we present outcomes with the use of this package for anesthesia and sedation analyzed specifically in 90 pediatric cases, including 53 pediatric cases which were included in the previous series but not separately analyzed there [17]. Further, we investigate the question as to what alternative approaches providers would have considered for performing painful procedures and surgeries in children if they had not had access to this package.

## Methods

**Fig. 1:**
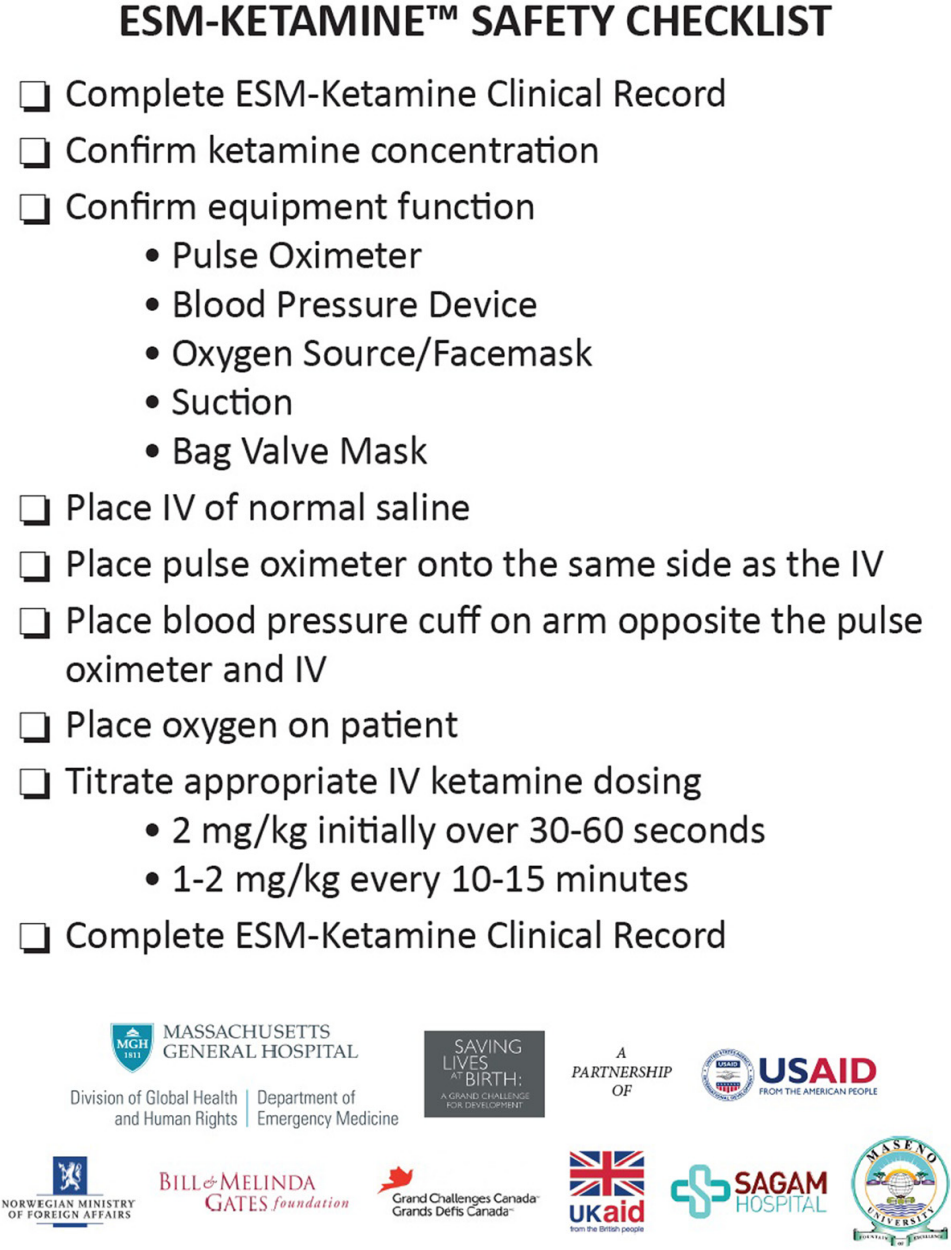
ESM-Ketamine safety checklist

As described previously, ESM-Ketamine is an evidence-based training package consisting of a 5-day hands-on training curriculum, clinical checklist (Fig. 1), wall chart, ketamine, and equipment (e.g., bag-mask ventilation device, pulse oximeter) [17]. This evidence-based program was developed by global health clinicians at Massachusetts General Hospital and Harvard Medical School and implemented in close partnership with the Kenya Ministry of Health and Maseno University School of Medicine (Maseno, Kenya).

Beginning in December 2013, local non-anesthetist clinicians within target facilities in rural Western Kenya were trained in the ESM-Ketamine package. The initial training roll out was focused on Sagam Community Hospital and took place during the first week of December 2013. Subsequent expansion trained providers at Mbita, Siaya, Maseno Mission, Yalla, and Nightingale Hospitals, with a total of 28 providers trained. Hospital selection criteria included facilities that have operating theaters and doctors who could perform surgical procedures. Health-care providers were selected by chief administrators from these hospitals and were trained on the ESM-Ketamine package. Training included modules on ketamine pharmacology, patient monitoring, oxygen delivery, newborn resuscitation, complication identification and intervention, and basic airway management (suction techniques, head-tilt and chin-lift, jaw thrust, two-person bagging). Participating clinicians were then invited to utilize the ESM-Ketamine package whenever they deemed a life-saving or life-improving surgery or procedure was necessary but were not able to obtain an anesthetist.

Data were collected prospectively by the clinician administering ketamine in all cases of ESM-Ketamine use at study facilities using a standardized ESM-Ketamine clinical record form. The form included elements such as patient age; patient weight; procedure performed; whether an anesthetist was called; and, if unavailable, details regarding why the anesthetist was unavailable. Clinicians also recorded complications attributable to the use of ketamine and adverse events at the time they occurred on the clinical record form. ESM-Ketamine quality assurance auditors collected completed ESM-Ketamine clinical record forms from the facilities on a weekly basis. Serious adverse events were defined as death, prolonged oxygen desaturation (more than 30 s) below 92 %, or any anesthesia-related injury, and minor adverse events were defined as brief oxygen desaturation (<30 s) below 92 %, hallucinations, and hypersalivation.

In this present study, we examined all ESM-Ketamine cases involving pediatric patients less than or equal to 18 years of age. The primary focus for analysis was patient safety, including patient outcomes and adverse events. In addition, we reviewed patient demographics and types of procedures completed using ESM-Ketamine. To determine how the option of ketamine sedation affected the management of pediatric cases, we interviewed ketamine providers who utilized the ESM-Ketamine package. Providers were asked how each patient in our series would have been approached if the ESM-Ketamine package had not been available, as well as how the provider might proceed with caring for a similar patient in the future.

This study was reviewed and approved in advance by the Kenya Ministry of Health, the institutional review board of Partners HealthCare (Massachusetts General Hospital, Boston, MA, USA), and the ethical review board of Maseno University School of Medicine (Maseno, Kenya).

## Results

### Cases

Over a 2-year period (December 13, 2013 to December 4, 2015), 90 life-saving or life-improving procedures were completed for 77 pediatric patients utilizing the ESM-Ketamine package. Of these, 32.2 % of cases were closed/open orthopedic reduction, 21.1 % were incision and drainage of an abscess, and 21.1 % were debridement and irrigation of burns. The remaining instances of ESM-Ketamine use in patients ≤18 years of age included obstetric/gynecologic procedures such as emergency cesarean section or repair of intrapartum perineal tear, foreign body removal, arthrocentesis, complex (non-gynecological) laceration repair, exploratory laparotomy, excision of mass, paracentesis, and circumcision (Table 1). The overall median dose of ketamine used was 2.0 mg/kg (range 1.4–8.3 mg/kg), and the median length of time needed to complete a procedure was 20 min (range 5–90 min). In 10 of the 90 cases, a second agent, either diazepam, tramadol, or diclofenac, was used in addition to ketamine. The cases that required the least amount of ketamine were arthrocentesis, closed orthopedic reduction, incision and drainage, circumcision, and foreign body removal. Longer, more complex procedures such as exploratory laparotomy, cesarean section, and orthopedic fracture reduction necessitated higher doses of ketamine. Among these seven more complex cases, one subject, undergoing open fracture reduction, experienced a minor adverse event in the form of hallucinations. The three cases of cesarean section took 35, 65, and 90 min, respectively. The two cases of exploratory laparotomy took 42 and 60 min, respectively. The two cases of orthopedic reduction lasted 5 and 35 min, respectively.

**Table 1:** Pediatric procedures and surgeries for which the ESM-Ketamine anesthesia package was utilized

Procedure	Number (%)	Median ketamine dose (and range) (mg/kg)	Median duration of procedure (and range) (min)
Arthrocentesis	4 (4.4)	2.0 (1.4–2.0)	20.0 (15.0–49.0)^b^
Cesarean section	3 (3.3)	5.5 (3.7–7.0)	65.0 (35.0–90.0)
Circumcision	1 (1.1)	2.8 (2.8–2.8)	13.0 (13.0–13.0)
Closed orthopedic reduction	27 (30.0)	2.0 (1.5–4.0)	18.2 (5.0–60.0)^b^
Complex laceration repair (non-gyn)	2 (2.2)	3.4 (3.0–3.8)	32.5 (25.0–40.0)
Debridement and irrigation of burns	19 (21.1)	2.9 (2.0–6.0)	20.0 (5.0–83.0)
Excision of mass	1 (1.1)	3.0 (3.0–3.0)	65.0 (65.0–65.0)
Exploratory laparotomy	2 (2.2)	6.0 (6.0–6.0)	51.0 (42.0–60.0)
Foreign body removal	6 (6.7)	3.0 (2.0–3.9)^a^	21.0 (11.0–30.0)
Gynecologic laceration repair	3 (3.3)	3.0 (2.0–4.9)	37.5 (15.0–60.0)^b^
Incision and drainage	19 (21.1)	2.0 (1.9–4.0)	20.0 (10.0–75.0)^b^
Open orthopedic reduction	2 (2.2)	5.6 (2.8–8.3)	20.0 (5.0–35.0)
Paracentesis	1 (1.1)	4.0 (4.0–4.0)	24.0 (24.0–24.0)
Total	90	2.0 (1.4–8.3)	20.0 (5.0–90.0)

^a^One case did not have a weight recorded, and so dose/kg could not be calculated

^b^10 cases had only one dose of ketamine so did not have total time recorded

### Patient demographics

Of the 77 pediatric patients treated with the ESM-Ketamine package, 36 (46.8 %) were female and 41 (53.2 %) were male. Ages ranged from 1 through 18 years, with a mean age of 10.6 years (Table 2).

**Table 2:** Age ranges and gender for patients in whom the ESM-Ketamine package was utilized

Age (years)	Gender, *N* (%)	
	Female	Male
0–1	0	0
1–4	8 (10.4)	6 (7.8)
5–12	11 (14.3)	21 (27.2)
13–18	17 (22.1)	14 (18.2)
Total	36 (46.8)	41 (53.2)

### Complications

In 15 (17 %) of the cases in which ESM-Ketamine was used, the patient experienced a minor adverse event. These reactions included hallucinations during recovery (nine patients), hypersalivation (four patients, one of which was given atropine), and fall in oxygen saturations <92 % for <30 s in two patients that self-resolved. There were no reported serious adverse events. None of the patients required cardiopulmonary resuscitation or bag-valve mask ventilation. None of the procedures were aborted or unable to be completed due to an adverse event.

### Provider survey responses

Follow-up surveys were conducted with 18 out of 23 providers who were involved in pediatric cases at four different hospitals to determine how they would have managed each case before they received the ESM-Ketamine package and training. Of the 28 total trained providers, five had not been involved in any pediatric cases at time of this study and five providers could not be reached as they were not available in person and their cellular phone numbers were no longer active. As such, responses were collected for 80 of the 90 pediatric cases (88.9 %) from 18 providers with the following results: 26 (32.5 %) of the procedures would have been done without any anesthesia or analgesia, 15 (18.75 %) would have been significantly delayed while the medical team waited for an anesthetist to become available, 13 (16.25 %) would have attempted referral to another facility that had anesthesia services, and 26 (32.5 %) would have been completed using an alternate form of anesthesia and/or analgesia. In this final category, in terms of which alternative drugs providers reported they would use, providers reported, in the absence of ketamine, they would use benzodiazepines (diazepam) plus pain medications (paracetamol, diclofenac, or ibuprofen) in 16 (62 %) of the cases, benzodiazepines alone in four (15 %), pain medicine alone in four (15 %), and local anesthesia in two (8 %). The only benzodiazepine available for use was diazepam, and the only pain medications available were paracetamol, diclofenac, and ibuprofen. One hundred percent of providers surveyed stated that they would use ketamine again for similar procedures in the future.

## Discussion

Our study supports the well-established safety record of ketamine for use in pediatric sedation and anesthesia. It corroborates other series that have demonstrated that this safety profile can be extended to the setting of limited resources with proper training and protocols for use. Furthermore, our study supports that the ESM-Ketamine package can extend anesthesia and sedation capabilities considerably for a wide variety of surgeries and painful pediatric procedures that might otherwise be performed with minimal or no analgesia/anesthesia or significantly delayed. Our sedation package was well-accepted by providers in a resource-limited setting, who report they would continue to use this package in future procedures. Thus, ESM-Ketamine represents an important tool in addressing the gap in pediatric anesthesia and analgesia that presently exists in many low-income countries.

Regarding ESM-Ketamine’s safety profile, 77 children underwent a total of 90 procedures using ketamine anesthesia in our study. Among these patients, there were no serious adverse events, defined as death, prolonged oxygen desaturation (>30 s), or any anesthesia-related injury. All adverse events reported were minor, with hallucinations representing the most commonly reported minor adverse event, followed by hypersalivation. These adverse event rates are comparable to previously reported rates for ketamine administered in children in both high-income and other low-income country settings [12, 16, 18].

The safety of the ESM-Ketamine package was enhanced by employing a simplified safety checklist with standardized ketamine dosing as well as including training in the management of potential complications. Airway events including laryngospasm, obstruction, and apnea are rarely reported with ketamine in the pediatric literature, and none occurred in our series of 90 cases [19, 20]. However, preparedness for the possibility of such events and management of airway emergencies is a critical component of the ESM-Ketamine training package.

To further reduce the risk of adverse events, the potential for medication error was minimized through the use of a standard dosing protocol, with an initial ketamine dose of 2 mg/kg administered strictly over a period of 30 to 60 s. This dose is within the range reported in the pediatric literature, where dosing generally ranges from 1 to 2 mg/kg [21, 22]. By standardizing initial dosing at 2 mg/kg for all ages and all procedures and then allowing up-titration in 0.5 mg/kg increments, our protocol both minimized risk of error while allowing for variation in patient responses to sedation, as well as length and type of procedure. Standard dosing also simplifies the regimen for the non-anesthetist providers for whom this training package was designed.

ESM-Ketamine anesthesia was utilized for a broad range of painful emergent and urgent pediatric procedures as well as more significant surgeries. As such, this package addresses two common gaps in the resource-limited setting: a void in options for analgesia for painful procedures as well as the paucity of available anesthesia services. The three most common procedures for which ketamine was used in our series were the following: closed orthopedic reduction, debridement of burns, and incision and drainage of abscesses. While none of these represent a major surgery, each of these represents a significantly painful procedure. The standard of care in children is to provide procedural sedation for such cases [20]. The use of the ESM-Ketamine package allowed for these procedures to be carried out without pain for the children in our study. In addition, our pediatric case series included two cases of exploratory laparotomy, two cases of open fracture reduction, and three cesarean sections. These surgeries, which require general anesthesia, were safely completed using ketamine when an anesthetist could not be present.

Further support for the importance of utilizing ESM-Ketamine to fill the gap in both analgesia and anesthesia for pediatric patients is evident in the responses to the provider surveys in our study. As reported by providers, without this package, approximately one third of the patients would have had their procedure completed without any analgesia or anesthesia. This would have likely led to considerable pain for the child, increased difficulty with completing the procedure, and psychological stress for the child and his/her caretaker(s). Another one third of the children in our study would have received only diazepam and/or paracetamol, ibuprofen, or diclofenac for pain control prior to procedure, which can lead to the same negative consequences. The remaining one third of patients would have had their procedure postponed for a variable duration, either due to the wait for an anesthetist to arrive or the time needed to attempt transfer of the patient to another facility, which may occur with significant delay and difficulty. In some of the cases, a delay in care could increase the risk of significant morbidity or mortality for the patient. Provider surveys support that ESM-Ketamine added an essential resource to prevent these adverse outcomes and provide safe, timely anesthesia and analgesia for pediatric patients undergoing surgeries and painful procedures in a limited-resource setting. Undoubtedly, these procedures could be performed to a higher quality with adequate analgesia and sedation than without it.

Our study had a number of limitations. Firstly, the sample size was relatively small. Given the infrequency with which adverse events occur with ketamine, a very large sample size would be required to understand how significant complications are managed in the rare circumstances in which they occur after ESM-Ketamine training. Secondly, only providers were surveyed for this study; patients and parents receiving this anesthesia package were not asked about their perception of the medication or whether they or their child experienced pain during the procedures. With regard to acceptance of the ESM-Ketamine package as obtained by interview with providers, there may have been a risk for social desirability bias. Efforts were made to mitigate this bias by explaining to participants that responses were confidential and anonymous and that the purpose of the interviews was to identify both successes and shortcomings of the intervention.

This study was conducted within four of the seven trained facilities in Western Kenya. Although these facilities represent differently sized facilities at different levels of the health system in Western Kenya, the findings of this study may not necessarily be generalizable to other low- and middle-income country settings. Further research examining the applicability of the ESM-Ketamine package in other resource-limited settings and countries would, therefore, be useful.

## Conclusions

Our study supports that ketamine’s well-established safety profile for pediatric anesthesia and sedation can be extended to a setting with limited resources with the ESM-Ketamine package which standardizes and packages training, drug delivery, and patient monitoring for non-anesthetist providers. This package, once implemented, can be used for select surgeries and painful procedures in children that might otherwise be performed with little or no analgesia and anesthesia or significantly delayed. It empowers non-anesthetist providers to be able to provide safe and effective analgesia and anesthesia to pediatric patients in emergent and urgent circumstances. The ESM-Ketamine package was well-accepted by providers trained in its use, evidenced by the reports they would continue to use this package in future procedures. As such, this package addresses a major gap in analgesia and anesthesia services for children in resource-limited settings. Further research should continue to monitor the safety of this package in children across a larger number of patients as well as explore the applicability and feasibility of ESM-Ketamine in additional low-income settings where the need to address the gap in pediatric anesthesia and analgesia remains great.

## References

[1] Hodges SM, Okello M, McCormick BA, Walker IA, Wilson IH (2007). Anaesthesia services in developing countries: defining the problems. Anaesthesia.

[2] Krauss BG (2006). Procedural sedation and analgesia in children. Lancet.

[3] Maman AK R, Zoumenou E, Gnassingbe K, Chobli M (2009). Anesthesia for children in Sub-Saharan Africa—a description of settings, common presenting conditions, techniques and outcomes. Pediatric Anesthesia.

[4] Meara JL, Hagander L, Alkire BC, Alonso N (2015). Global Surgery 2030: evidence and solutions for achieving health, welfare, and economic development. Lancet.

[5] Anderson RN, Chavez J, de Redon E, Burke T (2014). Defining the anesthesia gap for reproductive health procedures in resource-limited settings. Int J Gynceol Obstet.

[6] Dubowitz GD, McQueen KA (2010). Global anesthesia workforce crisis: a preliminary survey revealing shortages contributing to undesirable outcomes and unsafe practices. World J Surg.

[7] McQueen K (2010). Anesthesia and the global burden of surgical disease. Int Anesthesiol Clin.

[8] Zoumenou EG, Assouto P, Aboudoul-Fataou O, Lokossou T, Hounnou G, Aguemon AR, Chobli M (2010). Pediatric anesthesia in developing countries: experience in the two main university hospitalist of Benin in West Africa. Pediatric Anesthesia.

[9] Adudu P (2012). Anesthetic equipment, facilities and services available for pediatric anesthesia in Nigeria. Niger J Clin Pract.

[10] Hodges SH, Hodges AM (2000). A protocol for safe anaesthesia for cleft lip and palate surgery in developing countries. Anaesthesia.

[11] Bosenberg A (2007). Pediatric anesthesia in developing countries. Curr Opin Anaesthesiol.

[12] Bisanzo MN, Hammerstedt H, Dreifuss B, Nelson S, Charmberlain S, Kyomugisha F, Noble A, Arthur A, Thomas S (2012). Nurse-administered ketamine sedation in an emergency department in rural Uganda. Ann Emerg Med.

[13] Roelofse T (2010). The evolution of ketamine applications in children. Pediatric Anesthesia.

[14] Pederson LB (1993). Incidence and magnitude of hypoxaemia with ketamine in a rural African hospital. Anaesthesia.

[15] Strayer RN (2008). Adverse events associated with ketamine for procedural sedation in adults. Am J Emerg Med.

[16] Green SR, Lynch EL, Ho M, Harris T, Hestdalen R, Hopkins GA, Garrett W, Westcott K (1998). Intramuscular ketamine for pediatric sediation in the emergency department: safety profile in 1,022 cases. Ann Emerg Med.

[17] Burke TM, Altawil Z, DIckson A, Clark R, Okelo S, Ahn R (2015). A safe-anesthesia innovation for emergency and life-improving surgeries when no anesthetist is available: a descriptive review of 193 consecutive surgeries. World J Surg.

[18] Pitetti RS, Pierce MC (2003). Safe and efficacious use of procedural sedation and analgesia by nonanesthesiologists in a pediatric emergency department. Arch Pediatr Adolesc Med.

[19] Melendez EB (2009). Serious adverse events during procedural sedation with ketamine. Pediatr Emerg Care.

[20] Roback MW, Bajaj L, Bothner JP (2005). Adverse events associated with procedural sedation and analgesia in a pediatric emergency department: a comparison of common parenteral drugs. Acad Emerg Med.

[21] Dallimore DH, Short T, Anderson BJ (2008). Dosing ketamine for pediatric procedural sedation in the emegency department. Pediatr Emerg Care.

[22] Herd DA, Keene NA, Holford NH (2008). Investigating the pharmacodynamics of ketamine in children. Paediatr Anaesth.

